# Photoinactivation of Photosystem II in *Prochlorococcus* and *Synechococcus*

**DOI:** 10.1371/journal.pone.0168991

**Published:** 2017-01-27

**Authors:** Cole D. Murphy, Mitchell S. Roodvoets, Emily J. Austen, Allison Dolan, Audrey Barnett, Douglas A. Campbell

**Affiliations:** 1 Biochemistry and Chemistry, Mount Allison University, Sackville, New Brunswick, Canada; 2 Biology, Mount Allison University, Sackville, New Brunswick, Canada; 3 Michigan Technological University, Houghton, Michigan, United States of America; University of Hyderabad, INDIA

## Abstract

The marine picocyanobacteria *Synechococcus* and *Prochlorococcus* numerically dominate open ocean phytoplankton. Although evolutionarily related they are ecologically distinct, with different strategies to harvest, manage and exploit light. We grew representative strains of *Synechococcus* and *Prochlorococcus* and tracked their susceptibility to photoinactivation of Photosystem II under a range of light levels. As expected blue light provoked more rapid photoinactivation than did an equivalent level of red light. The previous growth light level altered the susceptibility of *Synechococcus*, but not *Prochlorococcus*, to this photoinactivation. We resolved a simple linear pattern when we expressed the yield of photoinactivation on the basis of photons delivered to Photosystem II photochemistry, plotted versus excitation pressure upon Photosystem II, the balance between excitation and downstream metabolism. A high excitation pressure increases the generation of reactive oxygen species, and thus increases the yield of photoinactivation of Photosystem II. Blue photons, however, retained a higher baseline photoinactivation across a wide range of excitation pressures. Our experiments thus uncovered the relative influences of the direct photoinactivation of Photosystem II by blue photons which dominates under low to moderate blue light, and photoinactivation as a side effect of reactive oxygen species which dominates under higher excitation pressure. *Synechococcus* enjoyed a positive metabolic return upon the repair or the synthesis of a Photosystem II, across the range of light levels we tested. In contrast *Prochlorococcus* only enjoyed a positive return upon synthesis of a Photosystem II up to 400 μmol photons m^-2^ s^-1^. These differential cost-benefits probably underlie the distinct photoacclimation strategies of the species.

## Introduction

*Synechococcus* and *Prochlorococcus* picocyanobacteria numerically dominate the open ocean phytoplankton community [1,2]. *Synechococcus* is most abundant in mesotrophic open ocean surface waters and coastal regions, and at lower abundances in nutrient-depleted areas [1,3]. Strains of *Synechococcus* tolerate a range of temperatures [4,5] and can thus extend to high latitudes. *Synechococcus* strains also vary in pigment composition allowing them to exploit different spectral niches [6,7]. *Prochlorococcus* is adapted to warm, stratified, oligotrophic tropical, and subtropical marine areas [1,8]. In areas where *Synechococcus* and *Prochlorococcus* co-occur, *Prochlorococcus* is typically more abundant. The two species have a high surface to volume ratio due to their small diameters of 0.5–0.8 μm for *Prochlorococcus* and 0.8–1.2 μm for *Synechococcus*. The smaller size of *Prochlorococcus* minimizes pigment package effects [9,10] giving it high optical absorption efficiency per pigment, that allows it to live deep in the water column, down to 200m [6,11]. This is optimal for photon capture but also leaves them vulnerable to photo-induced damage [12,13] since they lack the optical thickness for pigment screening [10]. Thus in addition to their ecological prominence *Synechococcus* and *Prochlorococcus* are intriguing minimal model species to study photosynthesis and photoacclimation in the absence of confounding biooptical complexities.

In oxygenic photoautotrophs pigment-proteins associated with Photosystem II (PSII) transfer excitation energy to the chlorophyll molecule P680, generating its excited state, P680* [14,15]. P680* then transfers the excited electron by reducing a phaeophytin (Phe) intermediate and drops to its oxidized ground state, P680^+^. The excited electrons are passed from Phe^-^ to Q_A_ and then to bound plastoquinone molecule Q_B_ which migrates to join the mobile plastoquinone (PQ) pool of the thylakoid membrane, eventually passing the electrons to the Cytochrome *b*_6*f*_ complex. The PQ pool can become saturated with electrons [4] if incoming excitation outruns downstream electron flow [16–18]. The P680^+^ radical is a strong oxidant and can cause irreversible damage to its surrounding proteins and pigments if it is not quickly reduced [19] by electrons from water within the oxygen-evolving complex of PSII [16–18].

*Synechococcus* and *Prochlorococcus* are characterized by different pigment-proteins to harvest light energy and protect PSII from photodamage. *Synechococcus* has an extra-membrane antenna complex, the phycobilisome, made up of phycocyanin rods that radiate from allophycocyanin core proteins. Core-membrane linker proteins bind the phycobilisome to the thylakoid membrane and transfer harvested light energy to chlorophyll *a* and carotenoids in the PSII complex [20–22]. The connectivity of the phycobilisome to the reaction centre can be regulated in some strains of *Synechococcus* [23,24] by an orange-carotenoid protein which mediates non-photochemical quenching of excitation [22,23,25,26].

*Prochlorococcus* instead uses intra-membrane prochlorophyte chlorophyll binding (Pcb) proteins as its light-harvesting complex. The Pcb proteins contain chlorophyll derivatives, di-vinyl chlorophyll *a* and di-vinyl chlorophyll *b*, that are specific to *Prochlorococcus* and have slightly different, blue-enhanced absorption spectra compared to the mono-vinyl chlorophyll *a* found in *Synechococcus* [27–29]. *Prochlorococcus* lacks the orange carotenoid protein but there is phenomenological evidence for some limited capacity for non-photochemical quenching even in *Prochlorococcus* [30] (K. Xu & D.A. Campbell, unpub.). Although non-photochemical quenching lowers the photochemical yield of PSII during periods of excess incident light, in cyanobacteria the non-photochemical quenching is relaxed within minutes upon return to more favorable light conditions [18,31]. In an alternate strategy cyanobacteria, including *Prochlorococcus*, can mediate pseudo-cyclic flows of electrons from PSII back to oxygen through multiple pathways [11,32–39] to control feedback inhibition of electron transport and thus lower the risk of reactive oxygen species production.

Light is essential for oxygenic photosynthesis, and oxygen is a product, but both have the potential to damage PSII protein subunits. The PsbA protein subunit of PSII is particularly susceptible to irreversible damage leading to the inactivation of the PSII complex [40]. The oxygen evolving complex of PSII can be inactivated by a photon in the UV or blue range directly absorbed by the Mn_4_Ca cluster [41]. Without the oxygen evolving complex extracting electrons from water, the P680^+^ radical cannot be rapidly reduced and may cause irreversible oxidative damage to the PsbA protein through uncontrolled oxidation of amino acids [42,43]. In samples dominated by this photoinactivation mechanism the rate constant of photoinhibition increases linearly with increasing irradiance [44,45]. Furthermore, Mn absorbance in the visible range is sufficient to account for the quantum, “per-photon”, yield of photoinhibition [18,41,46–48].

A second photoinactivation mechanism involves production of the reactive oxygen species singlet oxygen (^1^O_2_) [48–52] by a a normal triplet-state oxygen molecule reacting with a triplet excited state sensitizer molecule, the ^3^P680 state of chlorophyll, yielding ^1^O_2_ and the singlet ground state of the sensitizer molecule, P680 [48,53]. As electrons are then passed from one molecule to the next of the photosynthetic electron transport chain, short-lived radical intermediate states are generated. In PSII, if electron acceptors are available, electron transfer will happen before the radical intermediate can interact with anything else [18]. However, as the PQ pool becomes fully reduced, forward electron transport may be blocked at the level of Q_A_ or Q_B_ [18,50,54,55]. After primary charge separation a short lived ^3^[P680^+^Pheo^-^] charge pair is formed in its triplet ground state [56,57]. ^3^P680 thus occurs even under normal conditions and increases when forward electron flow slows through limitations downstream of Q_A_ or Q_B_ [58,59]. Triplet chlorophyll, ^3^Chl, a functional equivalent of ^3^P680, also occurs in the PSII antenna light harvesting complex in reactions independent of electron transport events [18,52,54,60,61] but proportional to the effective size of the photosynthetic antenna, which determines the average duration of excitation migration through the antenna.

Both the direct absorbance and inactivation of the Mn_4_Ca complex and reactive oxygen dependent photoinactivations occur [41,55,62,63] but show differing responses to incident light. The extinction coefficient (or target size) for a photon hitting the Mn_4_Ca complex is constant and so the probability of a single photon causing a photoinactivation should be independent of incident light level, while the rate of photoinactivation should show a simple linear dependence upon treatment light level. Furthermore, because Mn has higher absorbance in the blue and UV wavelength range and low absorbance in the red wavelength range, the probability of a photoinactivation is higher for a blue photon than for a red photon, no matter the spectral properties of the light harvesting antenna [18,41,64,65]. The probability of direct photoinactivation will, however, vary depending upon the optical screening and package effects around PSII which alter the achieved light incident upon the Mn_4_Ca complex. Since package effect varies with cell size larger cells show a lower probability of direct photoinactivation [65,66].

In contrast, the probability of generation of ^1^O_2_ shows a positive dependence upon increasing incident light level, which drives saturation of electron carriers out of PSII, leading to an increased probability of reactive oxygen species production [18]. Furthermore the antenna size of the PSII light harvesting complex will positively affect the per-photon probability of photoinactivation of PSII [67] since a larger antenna size supports greater photon capture, resulting in increased excitation pressure at a given light level [16,18,48].

**Fig 1:**
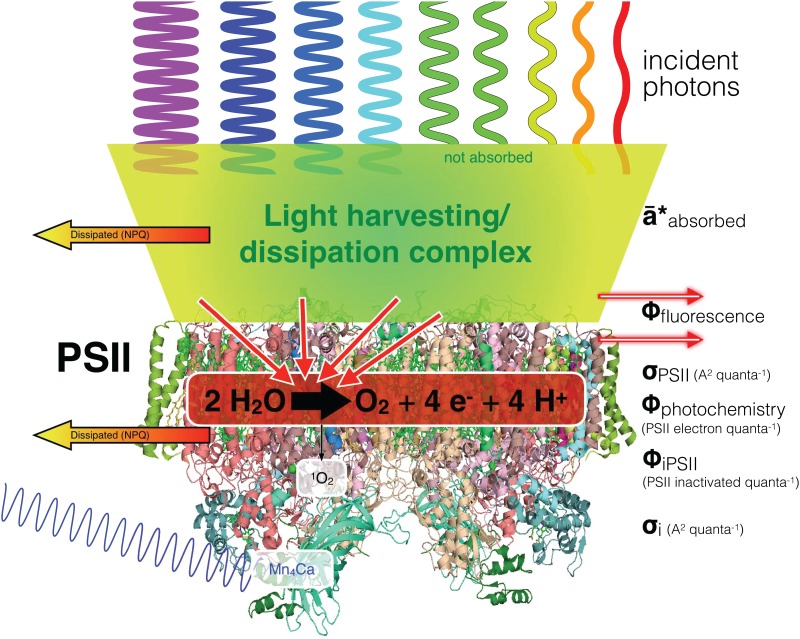
Conceptual overview of fates of photons in Photosystem II. Incident photons (downward waves) are differentially absorbed (ā*) depending upon the particular absorbance spectra of the antenna pigment protein complexes serving PSII, indicated as a green trapezoid to reflect the diversity of antenna pigments and protein structures across taxa. A variable fraction of the excitons is dissipated as heat (yellow-orange arrow, NPQ); a further small fraction of excitons are dissipated as fluorescence emitted from the antenna (red arrow). The remaining excitons are transferred into the inner antenna of the PSII reaction centre (downward red arrows). Of these excitons a small but variable fraction are again dissipated as fluorescence (red arrow) from the pigments of the reaction centre. The remaining excitons provoke PSII photochemistry (σ_PSII_; A^2^ quanta^-1^) leading to charge separation and electron transport with a variable yield (Φ_PSII_), or to fast recombinations resulting in heat emission (yellow-orange arrow, NPQ). If excitation is high relative to downstream electron transport excited intermediates can accumulate. This provokes production of singlet oxygen (^1^O_2_), which leads to an increase in the probability of PSII inactivation, herein parameterized Φ_i PSII_, a dimensionless yield for inactivation of a PSII by a photon delivered through the PSII antenna. Short wavelength photons can also be directly absorbed by the manganese cluster of the oxygen evolving complex (thin violet wave), leading to direct photoinactivation. This figure is based upon concepts reviewed in [18,44,52,62,68].

Fig 1 presents a schematic overview of these fluxes and fates of excitonic energy within PSII, drawing upon concepts reviewed elsewhere [18,44,52,62,68]. Incident photons are absorbed by antenna pigment proteins, whose spectral properties vary depending upon the taxon and acclimation state of the cell. Within the antenna complex both regulated and non-regulated processes [69] lead to dissipation of a variable fraction of the excitons as heat. A quantitatively minor fraction of excitons are also dissipated from the antenna as fluorescence, contributing to F_O_ level emission. The remaining, variable, fraction of excitons are transferred into the inner antenna of the PSII reaction centre, which is highly conserved across taxa. Of these excitons a small fraction are again dissipated as fluorescence from the pigments of the reaction centre, contributing to F_O_ level emission but also to the variable fluorescence F_V_ emitted by closed PSII centres. The remaining fraction of the excitons provoke PSII photochemistry, parameterized as σ_PSII_ (A^2^ quanta^-1^) [70–72], the effective absorption cross section for PSII photochemistry. PSII photochemistry can lead to charge separation and electron transport, parameterized as the yield Φ_PSII_, or to fast recombinations resulting in heat or delayed light emission [52]. If excitation delivery is high relative to downstream electron transport excited intermediates can accumulate [18]. This provokes production of singlet oxygen (^1^O_2_), which leads to an increase in the probability of PSII inactivation, herein parameterized as the yield for inactivation of PSII by a photon delivered through the PSII antenna, Φ_i PSII_. Φ_i PSII_ is thus predicted to increase with rising excitation pressure [62,68]. A few short wavelength photons can also be directly absorbed by the manganese cluster of the oxygen evolving complex, leading to photoinactivation whose probability per photon does not change with excitation pressure [41,48,62,73]. Under low to moderate blue light this direct path can dominate the effective absorption cross for inactivation of PSII on the basis of incident photons, parameterized as σ_i_ (A^2^ quanta^-1^) [44,66,74,75]. The relative magnitudes of the two categories of photoinactivation thus depends upon the spectral quality of the light relative to the antenna spectra of the organism, and upon the physiological state of the organism [76], which influences excitation pressure and capacities for reactive oxygen species detoxification.

Whatever the photoinactivation mechanism(s), as light level increases so will the rate of inactivation of PSII centers which must be countered through removal of inactive PSII proteins and synthesis of new ones to maintain photosynthetic activity. A damaged PsbA protein is degraded by the FtsH and/or Deg proteases [77–79], allowing the reconstitution and re-activation of the PSII center with a newly synthesized PsbA. Cells respond to increasing light by activating repair mechanisms including the up-regulation of *psbA* transcript levels, the gene encoding the PsbA protein [48,80–83]. Most cyanobacteria, although not *Prochlorococcus*, have small *psbA* gene families encoding two or more isoforms of the PsbA proteins with different photochemical properties [84–86] which are differentially induced depending upon irradiance and physiological state. The very repair cycle needed to regenerate PSII is itself strongly sensitive to inhibition by reactive oxygen [87–90] so net photoinhibition can result from both direct photoinactivation and from concomitant inhibition of the counteracting repair processes.

Photoinactivation and the regeneration of PSII through the PSII repair cycle imposes a significant metabolic burden upon the organism [91–94], which in turn places ecological limits upon their achieved niches and productivity [95,96]. Given the ecological importance of *Synechococcus* and *Prochlorococcus*, and their differential strategies to manage and exploit excitation [8,95–97] we sought to characterize the photoinactivation of PSII in these species. We used controlled culturing to grow representative strains of *Synechococcus* and *Prochlorococcus* under low and higher light. We then tracked their susceptibility to photoinactivation of PSII under a range of light levels, when counteracting PSII repair was blocked. We used a blue light treatment as an approximation of the blue-enriched light field in marine waters, and a red light treatment because the comparison with blue light was likely to be mechanistically informative [41,48,68]. We then used the composition of PSII and standard pathways to estimate the metabolic return on investment for repair or synthesis of a PSII, for each species under a range of light levels.

## Materials & Methods

### Culturing Conditions and Growth Rates

*Synechococcus sp*. WH8102 was grown in PCR-S11 media [98] while *Prochlorococcus marinus* MED4 was grown in Pro99 media [99,100]. Semi-continuous cultures for each species were maintained at low light (30 or 75 μmol photons m^-2^ s^-1^) or high light (260 μmol photons m^-2^ s^-1^) at 23–24°C under fluorescent lights with a 12:12 light:dark cycle with transitions from dark to light at 08:00 hours. A first round of semi-continuous cultures was grown in 50 mL tubes with plastic caps allowing airflow. A second round of semi-continuous cultures was grown in clear 6-well plates with each well containing 6.5 mL of culture. Data from both rounds of semi-continuous culturing was pooled for this manuscript after analyses of variance showed no significant effect of culture round upon measured variables (data not presented).

The growth of the cultures was tracked daily by fluorescence emission (Molecular Devices SpectraMax Gemini EM, Sunnyvale, California). *Prochlorococcus* was excited at 440 nm and the fluorescence emission was read at 680 nm. *Synechococcus* was excited at 550 nm and the fluorescence emission was read at 650 nm. For both genera the Relative Fluorescence Units (RFU) of their emission was tracked over elapsed time to calculate exponential growth rates for each sample using the equation:

$${\text{RFU}}_{t}={\text{RFU}}_{0}\;\times {\text{ e}}^{\mu \text{t}}$$ (1)

Where RFU_t_ is the fluorescence reading at elapsed time “t” and RFU_0_ is the fluorescence reading at time 0. The cultures were kept in exponential growth phase by 1 in 5 dilutions with fresh media every 5–7 days. Cultures were diluted and/ or harvested near the end of exponential phase to harvest the most biomass possible while the culture was still in exponential phase.

### Photosystem II Physiological Characterization

The photoinactivation treatments and measurements were carried out in Photon Systems Instruments FL3500 super-heads (Drasov, Czech Republic) with a lab-built aluminum plug for temperature control. For each treatment, a 2 mL sample was loaded into a cuvette with a micro stir-bar and plugged to hold the temperature at 23–24°C through circulation of cooling fluid through the aluminum plug. The super-head provided three capacities: firstly, application of sub-saturating flashlets of adjustable light level of 1.2 μs duration in red (655 nm LED) or blue (455 nm LED); secondly, detection of the fluorescence emission resulting from the flashlets; and thirdly, delivery of actinic light of adjustable intensity in red or blue (Table 1). We used the super-head flashlet capacity to perform fast repetition rate (FRR) chlorophyll fluorescence induction profiles, which were fit to a four parameter model (Table 2) to quantify PSII physiology [70] using PSIWORX-R data handling and fitting software implemented in the R environment (A. Barnett, http://sourceforge.net/projects/psiworx). The FRR induction curves were driven by a train of 40 flashlets of 1.2 μs each separated by 2.0 μs dark, for a cumulative flashlet train duration of 128 μs, shorter than the 200–500 μs for transfer of an electron from Qa to Qb, and almost 1 order of magnitude less than the subsequent ~1000 μs that it takes for the bound Qb pool of PSII to equilibrate with the plastoquinone pool [16,70]. FRR inductions were measured in the presence of background actinic light, and then again 2 s after the end of actinic light exposures.

**Table 1:** Treatments.

Species	Treatment Light Colour	Treatment Light Intensity (μmol photons m^-2^ s^-1^)
*Prochlorococcus marinus* MED4	Red	350, 500, 650, 700, 1200, 1300, 1400
*Prochlorococcus marinus* MED4	Blue	150, 250, 350, 400, 550, 600, 700, 900, 950,1250,
*Synechococcus sp*. WH8102	Red	350, 500, 650, 700, 750, 1000, 1200, 1500
*Synechococcus sp*. WH8102	Blue	150, 250, 300, 350, 400, 500, 550, 600, 700, 750, 950, 1050, 1200, 1250

**Table 2:** Fluorescence Parameters.

Parameter	Equation	Definition, units	Reference
F_0_		minimal fluorescence with PSII open	[107]
F_M_		maximal fluorescence with PSII closed	[107]
F_S_		fluorescence at an excitation level	[107]
F_M_′		maximal fluorescence with PSII closed at an excitation level	[107]
F_M_′2s		maximal fluorescence with PSII closed 2 s after excitation	[110]
F_0_′2s		minimal fluorescence with PSII open 2 s after excitation	[110]
F_0_′	F_0_/{(F_M_−F_0_)/F_M_ + F_0_/F_M_′2s}	minimal fluorescence with PSII open, estimated for cells under excitation, excluding cumulative influence of photoinactivation.	[108,111]
σ_PSII_		effective absorption cross section, Å^2^ photon^-1^, for PSII photochemistry	[70]
σ_PSII_′		effective absorption cross section, Å^2^ photon^-1^, for PSII photochemistry under excitation	[70]
σ_PSII_′2s		effective absorption cross section, Å^2^ photon^-1^, for PSII photochemistry 2 s after excitation	[110]
σ_i_	F_V_′ 2s/F_M_′ 2s = F_V_′ 2s/F_M_′ 2s_t = 0_ × e^(-σi × photon A2)^	target size, Å^2^ photon^-1^, for photoinactivation of PSII per cumulative incident photons, estimable across multiple excitation levels E	[44,66,74]
Φ_i PSII_	F_V_′ 2s/F_M_′ 2s = F_V_′ 2s/F_M_′ 2s_t = 0_ × e^(-Φi PSII × Photon PSII-1)^	Yield of PSII photoinactivations per photon delivered to PSII through the effective absorption cross section for PSII photochemistry σ_PSII_′	this work
1 –q_P_	1 –{(F_M_′–F_S_)/(F_M_′–F_0_′)}	Excitation pressure on PSII; balance between delivery of excitation and removal of electrons	[112]

### Whole-Cell Spectroscopy

**Fig 2:**
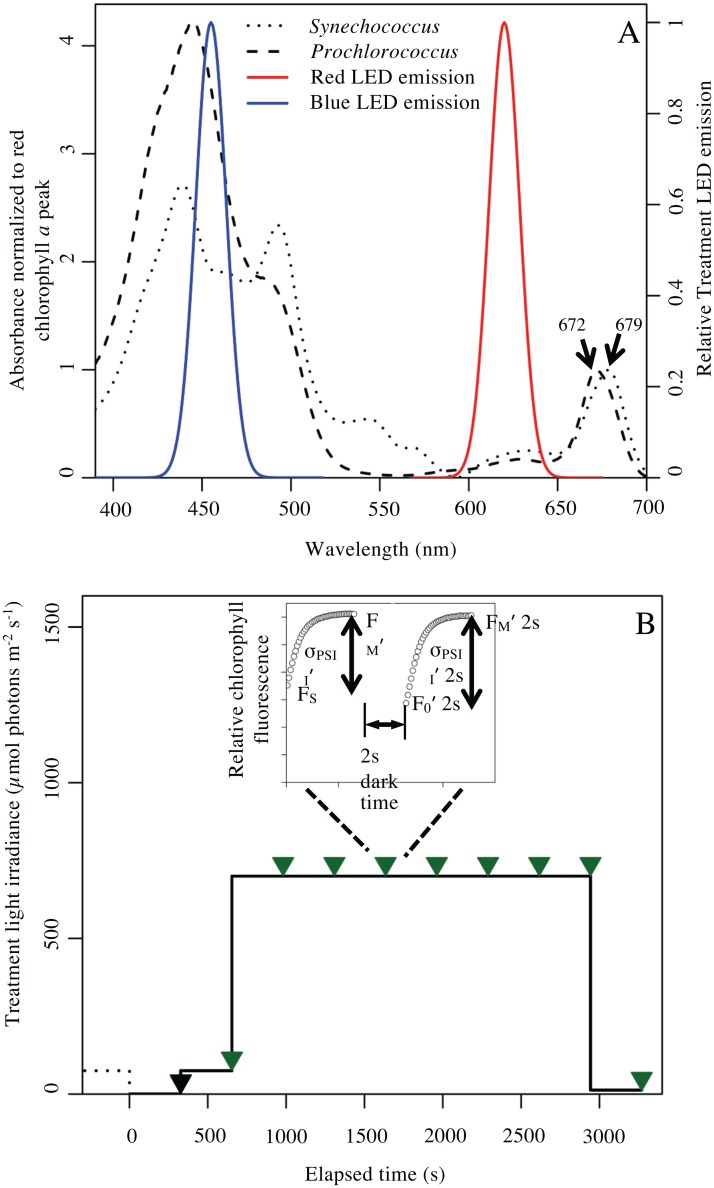
Cellular absorbance, light treatment color and protocol. **(A)** Whole Cell Absorbance Spectra of *Synechococcus sp*. WH8102 and *Prochlorococcus marinus* MED4. The relative emission spectra of the actinic blue and red LED used to induce photoinactivation (Fig 2B) in *Synechococcus* and *Prochlorococcus* are overlaid on the absorbance spectra, normalized to their red chlorophyll *a* peak. **(B)** Light intensity and measurement timing through the duration of a representative light treatment experiment. The pre-zero dotted line indicates the growth light for the culture; 75 in this representative figure; 30 or 260 μmol photons m^-2^ s^-1^ in other experiments. Each treatment time course then consisted of 10 sequential periods of 327 s, shown by the solid black line. The first 327 s period was in the dark, the second period was under the growth irradiance for the particular sample. The third to ninth treatment periods, shown here at 700 μmol photons m^-2^ s^-1^, were at irradiance and colour combinations shown in Table 1. The tenth period was a low light recovery phase of 20 μmol photons m^-2^ s^-1^. The black triangle indicates a Fast Repetition chlorophyll fluorescence induction measurement taken after the initial dark period, using 40 flashlets of 1.2 μs duration, spaced by 2 μs darkness, which cumulatively delivered a single turnover saturating flash over 128 μs. From this induction curve we used PSIWORX-R script to extract estimates for F_0_, F_M_ and σ_PSII_. Each green triangle thereafter indicates the timing of a chlorophyll fluorescence induction measurement taken after an illuminated period, represented in the inset above the treatment trace. At each measurement point we captured an induction applied in the presence of continuing actinic irradiance to extract estimates for F_S_, F_M_′ and σ_PSII_′. We then interrupted the actinic irradiance for 2 s of darkness to allow PSII centres to re-open, followed by another fluorescence induction to extract estimates for F_0_′, F_M_′ (2s) and σ_PSII_′(2s). The post-induction fluorescence relaxation phase of the full Fast Repetition and Relaxation fluorescence profile is omitted from this schematic diagram for clarity.

An absorbance spectrum was taken for each sample using an Olis CLARiTY DSPC spectrophotometer (Bogart, Georgia). The integrating, internally reflective DSPC cavity records accurate absorbance spectra from dilute suspensions of phytoplankton cells with high light scattering relative to low absorbance. Spectra were taken in the visible range, λ = 390–750 nm, at 1 nm intervals. Spectra of media blanks were subtracted from their respective genera and a conversion was performed to correct for chamber reflectivity using Fry’s Method [101]. This data transform takes the total absorbance spectra from the integrating, internally reflective cavity, and corrects it to the equivalent absorbance expected from a hypothetical 1 cm path length spectrophotometer cuvette measurement without scattering. Fig 2A compares typical absorbance spectra for *Prochlorococcus* and *Synehococcus* with the emission wavelength profiles of the LED lamps used to provide the light treatments.

### Photoinactivation Treatments

To measure photoinactivation the overlapping effects of the PSII repair cycle and non-photochemical quenching must both be considered [44,68]. Lincomycin at a final concentration of 500 μg mL^-1^, was used to inhibit prokaryotic ribosomes and thereby block the PSII repair cycle during the photoinactivation treatments [48,73,97,102]. Samples were subjected to light shift treatments of 10 consecutive periods of 327 s each, totaling 3270 s at a range of levels of blue or red treatment light (Table 1). The choice of 327 s was imposed by a limitation on the maximum time between sequential measurements in the software control of the fluorometer. Fig 2B presents a schematic overview of the photoinactivation treatment and measurement protocol. A FRR measurement was taken 2s after each illumination period and analyzed to extract photophysiological parameters (Table 2) including σ_PSII_′2s and F_V_′2s/F_M_′2s estimates corresponding to their respective preceding period.

The initial dark period allowed for the measurement of σ_PSII_ after relaxation of any non-photochemical quenching induced by growth conditions. F_V_′2s/F_M_′2s taken after 327 s under the sample growth light gave the maximal photochemical yield of PSII in the light acclimated state. F_V_′2s/F_M_′2s measured after each of 8 successive 327 s periods under the treatment light tracked the maximum photochemical yield in response to the light shift treatment. Finally, the terminal tenth low light period from 2943 to 3270 s then allowed the sample to reverse any changes caused by sustained NPQ which persisted through the 2s dark periods before measurements, but which could relax over the final 327 s under low light in the absence of protein synthesis, blocked by lincomycin. The influence of sustained NPQ on photochemical yield was then estimated as:

$$\text{Influence of Sustained NPQ }={\text{F}}_{\text{V}}\prime 2\text{s}/{\text{F}}_{\text{M}}\prime 2{\text{s}}_{\text{reverse}}-{\text{F}}_{\text{V}}\prime 2\text{s}/{\text{F}}_{\text{M}}\prime 2{\text{s}}_{\text{treatment}}$$ (2)

where Influence of Sustained NPQ was forced to be ≥0. The Influence of Sustained NPQ on photoinhibition were then corrected by adding the value to F_V_′2s/F_M_′2s measured after each treatment light interval. Therefore, we attributed any remaining decline in the maximum photochemical yield of (+) lincomycin samples to photoinactivation, which did not reverse in the absence of protein synthesis under the final 327 s of low light (Fig 2B). For the cultures in this study this correction had little influence on the subsequent curve fits, although in other taxa sustained NPQ is a significant factor confounding estimations of the photoinactivation of PSII [103].

### Quantitation of Photoinactivation

The corrected F_V_′2s/F_M_′2s values were then plotted against cumulative incident photons for the treatment light period.

$$\begin{array}{l} {\text{Cumulative Incident Photons photons m}}^{-2}\;=\;\left(6.022\;\times 10^{17}\;{\text{photons }\mu \text{mol}}^{-1}\right)\\ \times \;\Sigma_{\text{n}=2-9}\left({\text{Light Level of Period}}_{\text{n}}\;{\;\mu \text{mol photons m}}^{-2}{\text{s}}^{-1}\times \;{\text{Duration of Period}}_{\text{n}}\text{ s}\right) \end{array}$$ (3)

Where cumulative incident photons had units of quanta m^-2^, treatment light level had units of μmol photons m^-2^ s^-1^, and the duration of each period was a constant 327 s.

**Fig 3:**
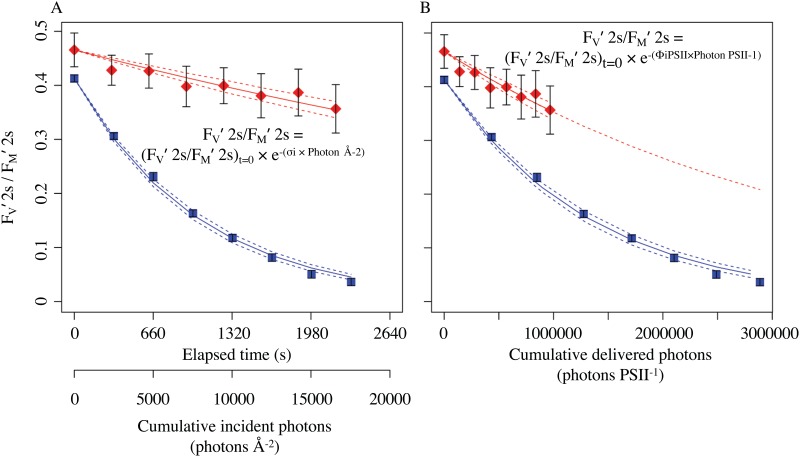
Representative photoinactivation treatment data. *Prochlorococcus marinus* MED4 was grown under 260 μmol photons m^-2^ s^-1^ and then treated under 1200 μmol photons m^-2^ s^-1^ of red light (red squares) or 1200 μmol photons m^-2^ s^-1^ of blue light (blue squares) following the protocol outlined in Fig 2A. For non-least squares non-linear modelling of the data (nlsLM, R) [104] each F_V_′ 2s/F_M_′ 2s derived from an individual Fast Repetition and Relaxation chlorophyll fluorescence induction after 2 s of darkness following actinic light conditions (Fig 2A, inset) was weighted by the inverse of its 95% confidence interval (plotted as error bars on the points) to account for variability in the precision of individual estimates of F_V_′ 2s/F_M_′ 2s. **(A)** The decay of the quantum yield of PSII (F_V_′ 2s/F_M_′ 2s) (Fig 2A, inset), plotted versus time and cumulative incident photons since the start of the treatment. The decay of F_V_′ 2s/F_M_′ 2s was fit (solid lines) to the annotated equation to extract σ_i_, a target size parameterization of the probability of an incident photon inducing photoinactivation of PSII. In these examples the σ_i_ was 1.65 × 10^−5^ Å^2^ PSII^-1^ under red light treatment (red diamonds) and 1.23 × 10^−4^ Å^2^ PSII^-1^ under blue light treatment (blue squares). 95% C.I. on the fit plotted as dotted lines. **(B)** The decay of F_V_′ 2s/F_M_′ 2s (solid lines) against cumulative photons delivered to PSII photochemistry, estimated as cumulative incident photons multiplied by the effective absorption cross section of the sample, σ_PSII_′ 2s. The decay is fit to the annotated equation to extract Φ_i PSII_, the probability of photoinactivation by a photon delivered to PSII through the antenna. The fitted values of Φ_i PSII_ were 2.8 × 10^−7^ PSII photon ^-1^ under red light treatment (red diamonds) and 7.2 × 10^−7^ PSII photon ^-1^ under blue light treatment (blue squares). 95% C.I. on the fit plotted as dotted lines.

A single-phase exponential decay curve was fit to the data using the following equation:

$${\text{F}}_{\text{V}}\prime 2\text{s}/{\text{F}}_{\text{M}}\prime 2\text{s}\;={\text{F}}_{\text{V}}\prime 2\text{s}/{\text{F}}_{\text{M}}\prime 2{\text{s}}_{\text{t}=0\;}\;\times \;{\text{e}}^{-\left(\sigma \text{i }\times \text{ Cumulative Incident Photons}\right)}$$ (4)

where σ_i_ is a target size parameterization [44,66,74] of the probability of a photoinactivation per incident photon with units of m^2^ quanta^-1^. Higher σ_i_ values indicate a higher probability of photoinactivation per incident photon, and a lower σ_i_ indicates a lower susceptibility to photoinactivation per incident photon. Fig 3A presents representative photoinactivation curves measured in the presence of lincomycin, under red or blue light treatments.

### Correcting σ_i_ for Photons Delivered to PSII

To correct for effects on σ_i_ caused by spectral or regulatory differences in delivery of excitation to PSII through the light harvesting antennae, susceptibility to photoinactivation was recalculated using cumulative photons delivered to PSII, as opposed to the previous σ_i_ calculation using cumulative photons incident upon the cells. A single-phase exponential decay was used to calculate the yield of photoinactivation of PSII on the basis of photons delivered to PSII (Φ_i PSII_):

$${\text{F}}_{\text{V}}\prime 2\text{s}/{\text{F}}_{\text{M}}\prime 2\text{s}\;={\text{F}}_{\text{V}}\prime 2\text{s}/{\text{F}}_{\text{M}}\prime 2{\text{s}}_{\text{t}=0\;}\;\times \;{\text{e}}^{-\left(\Phi \text{i PSII }\times \text{ Cumulative Delivered Photon PSII}-1\right)}$$ (5)

The parameter Φ_i PSII_ is a formally dimension-less yield (PSII inactivated photon^-1^ = quanta quanta^-1^) that expresses the number of PSII photoinactivation events per photon delivered to PSII through the effective absorption cross section for PSII photochemistry. We earlier expressed a conceptually similar concept [44] as the ratio of the effective absorption cross section for PSII photochemistry, σ_PSII_ to the effective absorption cross section for photoinactivation, σ_i_. Herein we used time-specific σ_PSII_′2s values measured every 327 s throughout the light-shift photoinactivation treatment to give the effective absorption cross section of PSII over that 327 s, in order to calculate the photons delivered to PSII over that period. Fig 3B presents representative photoinactivation curves measured in the presence of lincomycin, under red or blue light treatments plotted versus cumulative photons delivered to PSII.

### Testing the Response of Φ_i PSII_ to PSII Closure

If photoinactivation is driven by reactive oxygen by-products of excitation and electron fluxes through PSII [18,52,105] the susceptibility to photoinactivation should increase as excitation of PSII increases relative to electron transport away from PSII. To determine whether excitation pressure, the balance between excitation delivery to PSII, and electron flow away from PSII [106], indeed affected susceptibility to photoinactivation [18,52] we used the parameter 1 –q_P_ [107]

$$1\;-\;{\text{q}}_{\text{P}}\;=\;1\;-\;\left({\text{F}}_{\text{M}}\prime \;-\;{\text{F}}_{\text{S}}\right)/\left({\text{F}}_{\text{M}}\prime \;-\;{\text{F}}_{0}\prime \right)$$ (6)

where a value of 0 indicates all PSII are open and ready to accept excitation and a value of 1 indicates all PSII are closed and thus more susceptible to generation of reactive oxygen species. We calculated 1 –q_P_ for a given treatment after the initial 327 s exposure to the treatment light, as the value remained relatively unchanged thereafter with further treatment light exposure (data not shown). In these calculations we used a calculated F_0_′ (Table 2) [108,109].

### PSII Electron Transport versus Electron Equivalents to Recycle PSII

In order to determine the number of photochemical events performed per s by PSII under the treatment light, we used the effective absorption cross section of PSII under treatment light after 2 s of darkness (σ_PSII_′ 2s), the proportion of open PSII when exposed to treatment light (q_P_) and the intensity of the treatment light (I) [113,114]:

$$\text{e}-\;{\text{PSII}}^{-1}{\text{s}}^{-1}=\;\sigma_{\text{PSII}}\prime 2\text{s }\times {\text{ q}}_{\text{P}}\;\times \text{ I}$$ (7)

In order to determine the number of photoinactivation events per PSII per s, we multiplied the effective cross section for photoinactivation (σ_i_) (Å^2^quanta^-1^) by the intensity of the treatment light (I) (1 μmol photons m^-2^ s^-1^ = 6.022 × 10^−3^ quanta Å^-2^ s^-1^). We then estimated the investment in metabolic electron equivalents of ATP and reductant needed in order to either synthesize a complete *de novo* PSII (1.96 × 10^5^ e-/PSII), or to recycle most PSII protein subunits while proteolytically degrading and re-synthesizing the equivalent of new PsbA and PsbD proteins (5.77 × 10^3^ e-/(PsbA + PsbD)).

We arrived at these metabolic electron equivalent costs by summing the amino acid composition of the proteins of PSII [92,115] and accounting for the ATP costs of ribosomal protein synthesis [116–118], the electron and ATP costs for assimilation of CO_2_ and NO_3_^-^ to amino acids [119–121], and the ATP costs for FtsH protease turnover of PsbA and PsbD subunits [122,123]. We converted ATP costs to electron equivalents with the textbook ratio of 1.33 e^-^/ATP for respiratory electron transport. These approximations serve to place the cost of PSII inactivation on a common denominator for comparison with electron generation by PSII.

## Results & Discussion

### σ_i_ varies with Treatment Light Intensity and with Excitation Pressure on PSII

**Fig 4:**
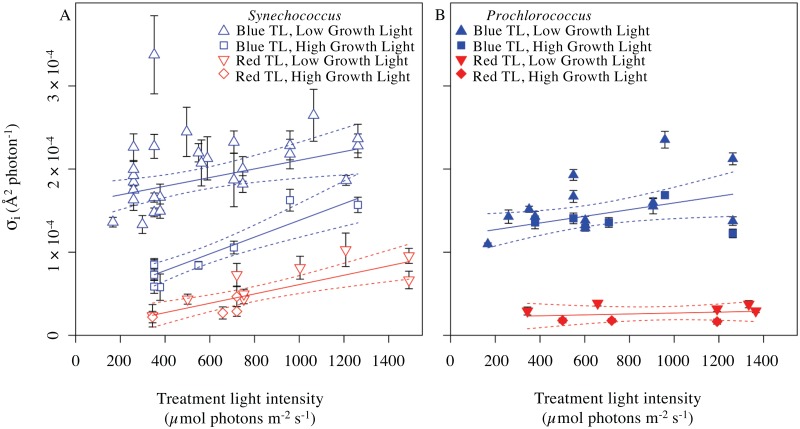
σ_i_ plotted against treatment light intensities. For regressions each σ_i_ derived from an individual treatment time course (ex. Fig 3A) was weighted by the inverse of its 95% confidence interval (error bars on the points) to account for variability in the precision of individual estimates of σ_i_. **(A)** σ_i_ from treatments of *Synechococcus sp*. WH8102 under a range of light levels. σ_i_ (Å^2^ photon^-1^) estimates derived from red light treatment (open red symbols) fell on a single regression against treatment light intensity (μmol photons m^-2^ s^-1^) for cultures grown under both low (red open inverted triangles) or high (red open diamonds) light; (solid red line, slope = 5.620 × 10^−8^ ± 1.212 × 10^−8^, intercept = 4.850 × 10^−6^ ± 1.027 × 10^−5^ (not significantly different from zero, p>0.05), R^2^ = 0.6616, dotted red lines denote 95% confidence intervals on the regression). σ_i_ estimates derived from blue light treatment (open blue symbols) fell on different regressions for cultures grown under low light (blue open triangles) (solid blue line, slope = 5.200 × 10^−8^ ± 1.814 × 10^−8^, intercept = 1.586 × 10^−4^ ± 1.146 × 10^−5^, R^2^ = 0.2401, dotted lines show 95% confidence intervals on the regression), or for cultures grown under high light (blue open squares) (slope = 1.008 × 10^−7^ ± 1.626 × 10^−8^, intercept = 3.749 × 10^−5^ ± 1.011 × 10^−5^, R^2^ = 0.846) (S1 Statistics). **(B)** σ_i_ from treatments of *Prochlorococcus marinus* MED4 measured under a range of treatment irradiances. σ_i_ estimates derived from red light treatment fell on a single regression (solid red line, slope not significantly different from zero, p>0.05; intercept = 2.130 × 10^−5^ ± 9.023 × 10^−6^, R^2^ = 0.05971, dotted red lines denote 95% confidence intervals) for cultures grown under either low (red inverted triangles) or high (red diamonds) light. σ_i_ estimates derived from blue light treatment fell on a single regression (solid blue line, slope = 4.013 × 10^−8^ ± 1.714 × 10^−8^, intercept = 1.191 × 10^−4^ ± 1.214 × 10^−5^, R^2^ = 0.2437, dotted blue lines denote 95% confidence intervals) for cultures grown under low (blue triangles) or high (blue squares) growth light (S2 Statistics).

σ_i_ estimates were derived from individual photoinactivation time courses in the presence of lincomycin (Fig 3A) for combinations of species, growth light level, treatment light colour and treatment light intensity (Table 1). Fig 4 presents these estimates of σ_i_ plotted versus the treatment light intensity. For fitting of regressions each σ_i_ estimate was weighted by the inverse of its 95% confidence interval, plotted as error bars on the points, to account for variability in the precision of individual estimates of σ_i_ (Fig 3A).

Under red light σ_i_ was low for both *Synechococcus* and *Prochlorococcus*. For *Synechococcus* the Y intercept of the regression of σ_i_ versus treatment light intensity was not significantly different from 0, but there was a significant increase in photoinactivation with increasing intensity of red light treatment. In *Prochlorococcus* the Y intercept was small and there was no significant increase in σ_i_ with increasing intensity of red light treatment. Under blue light treatment σ_i_ was significantly higher than under red light, consistent with expectations [41,48,65,68,124,125]. *Synechococcus* cultures grown under high light fell on regression with intercept and slope distinct from *Synechococcus* cultures grown under lower light. When treated under blue light the *Prochlorococcus* low light and higher light grown cultures all fell on a common regression but there was an increase in σ_i_ with increasing intensity of blue light treatment.

**Fig 5:**
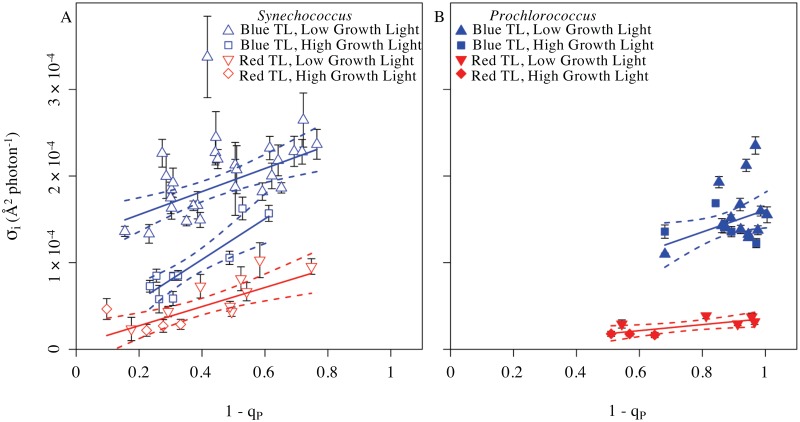
σ_i_ versus excitation pressure on Photosystem II. σ_i_ plotted against excitation pressure, measured as 1—q_P_ after 300 s of treatment light exposure. Under red or blue treatment light, q_P_ showed remained nearly steady from 327 s onwards (data not shown) so the plotted values for 1—q_P_ were taken after the first period of 327 exposure to treatment light. For regressions each σ_i_ derived from an individual treatment timecourse (Fig 3A) was weighted by the inverse of its 95% confidence interval (error bars on the points) to account for variability in the precision of individual estimates of σ_i_. **(A)** σ_i_ from *Synechococcus sp*. WH8102. σ_i_ estimates derived from red light treatments fell on a single regression (solid red line, slope = 1.103 × 10^−4^ ± 2.670 × 10^−5^, intercept not significantly different from zero, p>0.05; R^2^ = 0.6079, dotted red lines denote 95% confidence intervals on the regression). for *Synechococcus* cultures grown under low (red open inverted triangles) or high (red open diamonds) growth light. For σ_i_ estimates derived from blue light treatment, low light grown cultures (blue open triangles) fell upon a regression (solid blue line, slope = 2.349 × 10^−4^ ± 4.437 × 10^−5^, intercept = 9.742 × 10^−6^ ± 1.540 × 10^−5^, R^2^ = 0.3813, dotted lines show 95 % confidence intervals), while the high light grown cultures (open blue squares) fell upon a different regression (solid blue line, slope = 1.332 × 10^−4^ ± 3.326 × 10^−5^, intercept not significantly different from zero, p>0.05; R^2^ = 0.8001, dotted lines show 95 % confidence intervals on the regression) (S3 Statistics). **(B)** σ_i_ from *Prochlorococcus marinus* MED4. σ_i_ estimates derived from red light treatment (low light grown cultures, red inverted triangles; high light grown cultures, red diamonds) fell on a single regression (solid red line, slope = 3.484 × 10^−5^ ± 1.222 × 10^−5^, intercept not significantly different from zero, p>0.05;, R^2^ = 0.5755, dotted red lines denote 95 % confidence intervals). σ_i_ estimates derived from blue light treatment (low light grown cultures, blue triangles; high light grown cultures, blue squares) fell on a single regression (solid blue line, slope = 1.262 × 10^−4^ ± 5.667 × 10^−5^, intercept not significantly different from zero, p>0.05; R^2^ = 0.2259, dotted blue lines denote 95 % confidence intervals) (S4 Statistics).

The absorbance spectra of the cultures varied with species (Fig 2A) and with growth light (data not shown). The photosynthetic physiologies of the species are also distinct with evidence for down-stream limitations on the rate of electron transport away from PSII in *Prochlorococcus* when compared to *Synechococcus* [11,126,127]. Therefore a given treatment light level or colour could have differing functional implications for the cell depending upon species and prior light regime [76]. We therefore decided to replot the σ_i_ estimates versus the excitation pressure upon PSII imposed by each given combination of treatment light intensity and colour (Fig 5A and 5B). These replots show patterns very similar to the plots of σ_i_ versus treatment light intensity (Fig 4A and 4B), except that under red light treatment *Prochlorococcus* shows a small positive response of σ_i_ to increasing excitation pressure.

The Y intercepts on these plots represent the inherent photoinactivation potential of an incident photon in the absence of any excitation pressure, and therefore in the absence of any electron transport or risk of reactive oxygen generation by PSII or related electron flows. Under red light treatment both *Synechococcus* and *Prochlorococcus* show Y intercepts not significantly different from 0 for these plots of σ_i_ versus increasing excitation pressure. Thus at the Y intercept, in the absence of electron transport, red photons show no detectable intrinsic toxicity to PSII. The low level of photoinactivation under red light is thus quantitatively attributable to by-products of electron transport through PSII.

Under blue light treatments *Synechococcus* grown under lower light, and *Prochlorococcus*, σ_i_ shows Y intercepts significantly higher than 0, demonstrating an intrinsic toxicity of blue light to PSII [48,62,68,124], even in the absence of electron flow and its concomitant reactive oxygen species byproducts. *Synechococcus* grown under higher light showed low (Fig 4A) or insignificant (Fig 5A) Y intercepts for σ_i_, and thus showed a lower intrinsic susceptibility to blue light photoinactivation, which could relate to expression of alternate isoforms of the PsbA protein [84–86] in *Synechococcus*.

The rising slopes of σ_i_ with increasing excitation pressure upon PSII represent the outcome of a complex balance among multiple paths [18,52,105] of potential reactive oxygen species production related to electron fluxes (Fig 1) and the counteracting detoxification mechanisms whose presence and induction can vary both with species and with prior growth conditions [8,76].

### Φ_i PSII_ versus Excitation Pressure on PSII to Reconcile Species and Growth Light Effects

**Fig 6:**
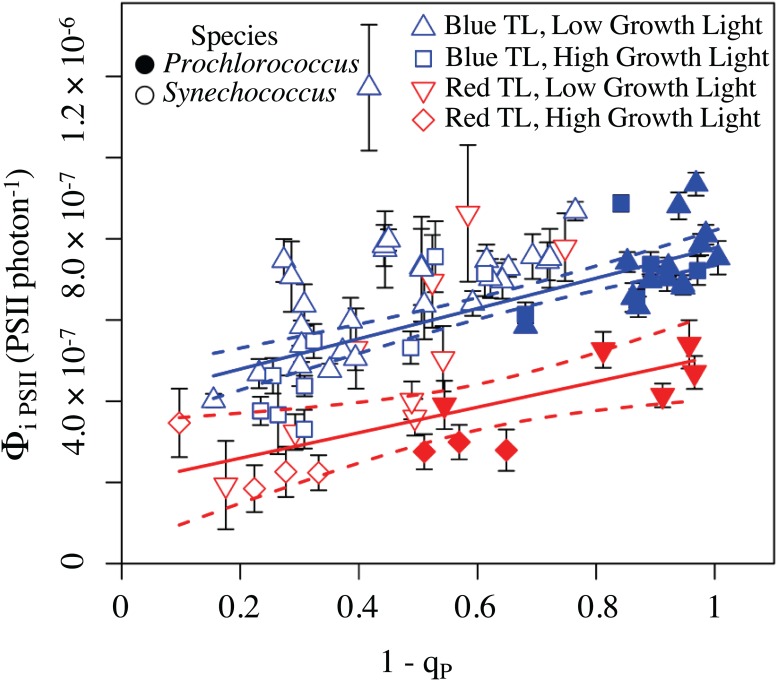
Φ_i PSII_ versus excitation pressure on Photosystem II. *Synechococcus sp*. WH8102 (open symbols) and *Prochlorococcus marinus* MED4 (closed symbols) fell on a common regression for Φ_i PSII_ (PSII photon^-1^) measured under red light treatment (solid red line, slope = 3.142 × 10^−7^ ± 1.110 × 10^−7^, intercept = 1.968 × 10^−7^ ± 7.308 × 10^−8^, R^2^ = 0.2968, dotted red lines denote 95% confidence intervals). *Synechococcus sp*. WH8102 and *Prochlorococcus marinus* MED4 also fell on a common regression Φ_i PSII_ measured under blue light treatment (solid blue line, slope = 3.731 × 10^−7^ ± 4.921 × 10^−8^, intercept = 4.046 × 10^−7^ ± 3.458 × 10^−8^, R^2^ = 0.5156, dotted red lines denote 95% confidence intervals on the regressions). Species had no statistically significant effect on the regressions of Φ_i PSII_ versus excitatation pressure, nor did low (circles) versus high (squares) growth light when either species or growth light was including in a linear model of the data as a binary interaction term, using ‘lm’ in R [128] (S5 Statistics).

We next asked whether the photoinactivation patterns could be reconciled by considering the yield of photoinactivation on the basis of photons delivered to PSII via the light harvesting antenna. We therefore estimated a new parameterization, Φ_i PSII_ to express the yield of photoinactivation of PSII relative to photons driving PSII photochemistry, as measured by the effective absorption cross section for photochemistry, σ_PSII_. For plots of Φ_i PSII_ versus excitation pressure (Fig 6) *Synechococcus* and *Prochlorococcus* from both low and higher prior growth lights all fell on a common regression under red light treatment, and on a common regression for blue light treatment. The two treatment lights gave equivalent slopes with increasing excitation pressure, but were distinguised by a higher Y intercept for blue light treatments. Thus the intrinsic toxicity of blue light, likely through direct photoinactivation of the manganese cluster of PSII [41,48] manifests as a strong potential for photoinactivation by blue photons even when electron flow through PSII is negligible. With rising excitation pressure both blue and red light drive comparable increases in photoinactivation, when expressed on the basis of excitation actually delivered to PSII photochemistry, rather than on the basis of incident photons. The relative importance of these distinct photoinactivation paths [62,68] will depend upon cellular absorbance spectra and the light level, but more particularly upon the physiological excitation pressure upon PSII, a proxy for the probability of generation of reactive oxygen species. Under physiologically low to moderate levels of light direct photoinactivation by blue light can dominate, and can be parameterized as a simple target size [66,73–75]. This conditions would often prevail in marine systems. As excitation pressure rises either through increasing light in near-surface environments or through factors that restrict the down-stream removal of electrons from PSII [39,106] the reactive oxygen species -dependent photoinactivation paths related to electron transport will increase and even predominate. These patterns become apparent across spectrally diverse species with different growth histories by expressing photoinactivation on the basis of photons delivered to PSII, plotted versus excitation pressure, rather than the treatment light level *per se*.

For regressions each Φ_i PSII_ derived from an individual treatment time course (Fig 3B) was weighted by the inverse of its 95% confidence interval (error bars on the points) to account for variability in the precision of individual estimates of Φ_i PSII_.

### The Return on Investment for Photosystem II in the Face of Photoinactivation

**Fig 7:**
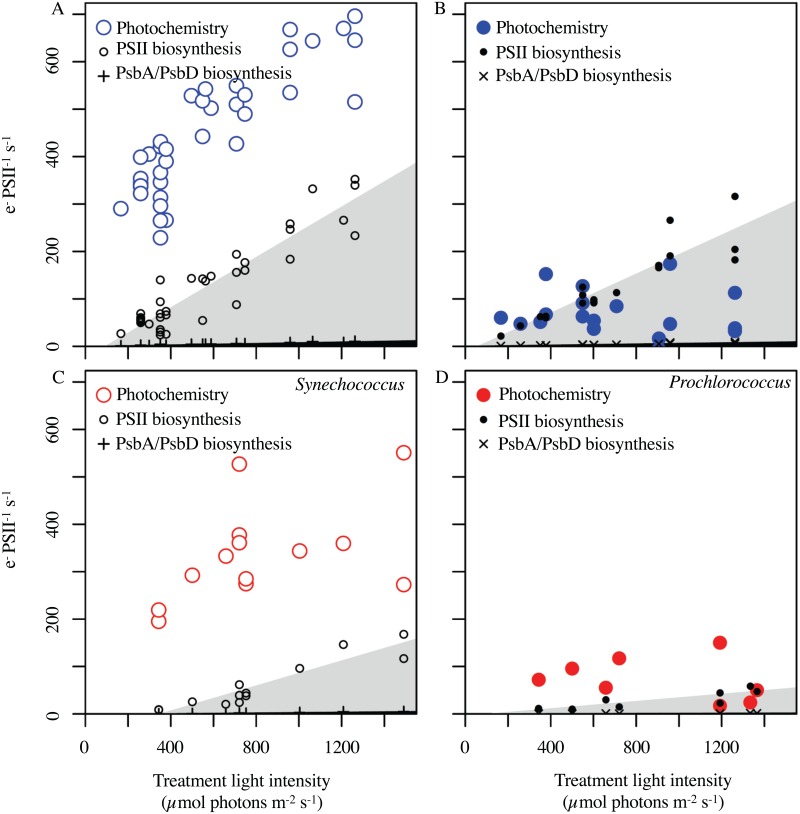
Rates of electron transfer through Photosystem II versus Treatment Light intensity, compared to electron equivalent cost for Photosystem II synthesis or recycling. Photochemical events (larger circles) were estimated as σ_PSII_′ 2s × q_P_ × E. Photoinactivation eventswere estimated as σ_i_ × E and multiplied by the e^-^ equivalent cost for biosynthesis of PSII (smaller black circles; shaded grey segment) or by the e^-^ equivalent cost for turnover of PsbA/PsbD (+ or ×; black segment along X axes). **(A)** *Synechococcus* under blue light. **(B)** *Prochlorococcus* under blue light. **(C)** *Synechococcus* under red light. **(D)** *Prochlorococcus* under red light.

The inactivation of PSII and the counteracting repair processes impose a significant, and variable, metabolic burden upon photoautotrophs [91–94,119] that interacts with nutrient supply [129,130] and with cell size [66]. We thus sought to use the optically simple *Synechococcus* and *Prochlorococcus* to compare the photochemical return on investment from a PSII, to the costs for net biosynthesis of a PSII from inorganic precursors, or to the costs to degrade and replace the PsbA and PsbD subunits of the complex (Fig 7). Our present analyses is restricted to nutrient replete cultures, but nutrient limitation likely interacts with these processes [131,132]. Photochemical events through PSII were estimated as (σ_PSII_′ 2s) × q_P_ × E. Photoinactivation events were estimated as σ_i_ × E and then multiplied by the e^-^ equivalent cost for biosynthesis of PSII or by the e^-^ equivalent cost for turnover of PsbA/PsbD. PSII Electron transport at a given treatment light level was consistently higher in *Synechococcus* than in *Prochlorococcus*, consistent with larger σ_PSII_ and greater down stream capacity for electron fluxes in *Synechococcus* [127]

Fig 7 plots a narrow black segment along the X axes to show the low metabolic cost needed to repair a photoinactivated PSII through degradation and re-synthesis of new PsbA/PsbD proteins. Across the tested range of blue or red treatment light levels *Synechococcus* PSII electron transport stayed well above this cost to repair a photoinactivated PSII, so PSII repair was always a metabolically viable strategy for our *Synechococcus* cultures, at least when growing under nutrient repletion. In contrast in *Prochlorococcus* much lower PSII electron transport, possibly limited by downstream metabolic capacity [127] means that the photochemical return from a PSII drops to levels similar to the metabolic cost to repair a PSII at light levels of ~1200 μmol photons m^-2^ s^-1^ for red or for blue treatment lights. The wider grey segment shows the metabolic cost for net biosynthesis of a new PSII from inorganic precursors at a rate sufficient to counter photoinactivation. Again, *Synechococcus* enjoys a positive net return on newly synthesized PSII up to and beyond 1200 μmol photons m^-2^ s^-1^, under both blue and red treatment lights, at least when growing under nutrient repletion and near-optimal temperatures. In marked contrast for *Prochlorococcus* under blue light the net cost to biosynthesize a new PSII exceeds the anticipated photochemical return from the new PSII at light levels above ~300 μmol photons m^-2^ s^-1^. Under the informative but less ecophysiologically relevant red light the lower rates of photoinactivation mean *Prochlorococcus* enjoys a positive return upon a new PSII up to light levels of ~1200 μmol photons m^-2^ s^-1^. These contrasting patterns in *Synechococcus* and *Prochlorococcus* support findings [8] that *Synechococcus* tends towards active mechanisms to cope with increasing excitation. In contrast *Prochlorococcus* tends to merely endure transient exposure to excess excitation and might trend towards mixotrophy under some conditions [133] when maintaining photosynthesis becomes untenable. These contrasting patterns also underlie findings of different excitation tolerance thresholds for the different picocyanobacteria [95,96], and their niche partitioning [97].

## Supporting Information

S1 Dataset Data File. Data records underlying Figs 4,5,6 and 7, supporting the key conclusions of the manuscript.
(ZIP)

S1 Statistics Statistics for Fig 4A. (TXT)

S2 Statistics Statistics for Fig 4B. (TXT)

S3 Statistics Statistics for Fig 5A. (TXT)

S4 Statistics Statistics for Fig 5B. (TXT)

S5 Statistics Statistics for Fig 6. (TXT)
